# Short- and long-term outcomes of 486 consecutive laparoscopic splenectomy in a single institution

**DOI:** 10.1097/MD.0000000000025308

**Published:** 2021-04-02

**Authors:** Xiaowei Fu, Zhengjiang Yang, Shuju Tu, Wanpeng Xin, Haiming Chen, Xueming Li, Yong Li, Weidong Xiao

**Affiliations:** a Department of General Surgery; b Department of Emergency Surgery, The First Affiliated Hospital of Nanchang University, Nanchang, China.

**Keywords:** hand-assisted, laparoscopic splenectomy, morbidity, postoperative outcomes

## Abstract

Since its introduction in 1991, laparoscopic splenectomy (LS) has become the gold standard in elective spleen surgery in many centres. However, there still lack the report of long-term outcomes of LS with the large-scale cases. The aim of the present study was to analyze the short- and long-term outcomes of LS in a single institution over 16 years, and to compare the perioperative outcomes of totally laparoscopic splenectomy (TLS) and hand-assisted laparoscopic splenectomy (HALS) for splenomegaly.

Between November 2002 and December 2018, 486 consecutive patients undergoing elective LS were enrolled in this study, including 222 TLS and 264 HALS. The intraoperative, postoperative, and follow-up data were retrospectively analyzed.

The 5 most common indications were hypersplenism (71.0%), immune thrombocytopenia (14.8%), splenic benign tumor (4.5%), splenic cyst (2.9%), and splenic malignant tumor (2.9%). The mean operative time, intraoperative blood loss, and length of stay were 149.4 ± 63.3 minutes, 230.1 ± 225.1 mL, and 6.7 ± 3.2 days, respectively. The morbidity, mortality, reoperation, and conversion rate were 23.0%, 0, 0.4%, and 1.9%, respectively. Portal vein system thrombosis (PVST) was the most frequent complication with an incidence of 19.8%. The incidence of PVST in HALS was higher than that in TLS (23.9% vs 14.9%, *P* = .013). Compared with TLS, HALS had a shorter operative time (*P* = .000), lower intraoperative blood loss (*P* = .000), comparable conversion rate (*P* = .271), and morbidity (*P* = .922) for splenomegaly > 17.0 cm. During the follow-up period, the overall respond rate for immune thrombocytopenia was 77.8%, and the esophagogastric variceal bleeding rate was 6.9% in 320 patients with hypersplenism secondary to hepatic cirrhosis.

LS is a safe, feasible, and effective procedure with satisfactory short- and long-term outcomes. HALS is a reasonable technique in patients with massive spleens.

## 1 Introduction

In 1991, Delaitre and Maignien^[1]^ reported the first successful laparoscopic splenectomy (LS). Following that inspiring initial experience, laparoscopic approaches to splenic surgery have been demonstrated to be safe and feasible by numerous cases. Compared with open splenectomy (OS), LS had advantages of less estimated blood loss, less requirements for transfusion, lower postoperative morbidity rate, faster recovery, and improved quality of life.^[2,3]^ LS is primarily used for elective resection in patients with benign spleen diseases, including primary hematological diseases, immune thrombocytopenia (ITP), spleen hamartoma, and hypersplenism.

With rapidly advancing in laparoscopic techniques, totally laparoscopic splenectomy (TLS) is nowadays considered as the gold standard for normal to moderately enlarged spleens. However, the adoption of TLS in patients with massive splenomegaly secondary to liver cirrhosis and portal hypertension introduces more difficulties than OS because of the enormous size of the spleen and existence of varicose vessels and coagulation disorders.^[4]^ Several studies have demonstrated that TLS for massive splenomegaly had longer operation time, more blood loss, and higher conversion rate than TLS for normal-sized spleens.^[5,6]^ In 1995, Kusminsky et al^[7]^ introduced the technique of hand-assisted laparoscopic splenectomy (HALS). This technique allows hand-assisted manipulation and dissection of the spleen, manual control of large vessels, and removal of an intact spleen through the hand port. Therefore, the introduction of hand-assisted technique has broadened the scope of LS to massive splenomegaly.

Although LS is widely performed in many centers, there still lack the report of long-term outcomes of LS with large-scale cases, and controversy still remains regarding the best approach for patients with massive splenomegaly. The first TLS and HALS of our institution were successfully performed in November 2002 and March 2006, respectively. Up to now, we had completed more than 500 cases of elective or emergency LS. This has inspired us to present our experience of LS over a 16 years period. The aim of the present study was to analyze the short- and long-term outcomes in a series of 486 elective LS (including TLS and HALS) from a single institution. Furthermore, we compared the perioperative outcomes of TLS and HALS for patients with splenomegaly, which maximum diameter of spleen greater than 17.0 cm.

## 2 Materials and methods

### 2.1 Study design

A retrospective cohort study, using a prospectively collected database, included all consecutive patients undergoing elective LS (including TLS and HALS) in our institution from November 2002 to December 2018. The inclusion criteria of this study were:

(1) patients with primary or secondary spleen diseases who underwent elective LS in our institution;
(2) Child-Pugh class A or B;
(3) no organic lesions in the heart, lung, kidney, or other important organs.

Patients who could not tolerate pneumoperitoneum had severe disease in other systems that affected their daily life, preoperative imaging examination has found thrombus in the portal vein system, underwent emergency LS due to splenic rupture or without complete clinical data were excluded. Written informed consent was obtained from each participant in accordance with the Declaration of Helsinki. The Ethics Review Board of the first affiliated hospital of Nanchang University approved this study (no. 2020B0017).

### 2.2 Patients

**Figure 1:**
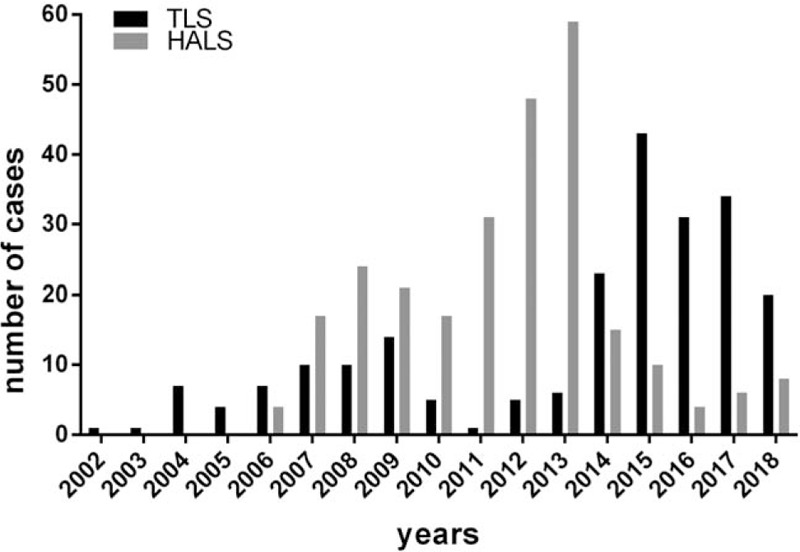
Number of cases done yearly.

A total of 486 patients were enrolled in this retrospective study, including 222 (45.7%) TLS and 264 (54.3%) HALS. The annually number of cases was shown in Figure 1. There were 257 men and 229 women with a median age of 43.4 ± 12.3 (range 12–75) years. The 5 most common indications were hypersplenism (71.0%), ITP (14.8%), splenic benign tumor (4.5%), splenic cyst (2.9%), and splenic malignant tumor (2.9%). The primary diseases of hypersplenism included hepatitis B virus (HBV)-related hepatic cirrhosis (n = 316), alcoholic cirrhosis (n = 4), hepatitis C virus (HCV)-related cirrhosis (n = 7), schistosomiasis cirrhosis (n = 12), and mixed cirrhosis (n = 6). All patients’ characteristics, surgical features, and intraoperative and postoperative outcomes were retrospectively reviewed.

### 2.3 Operative technique

In TLS, patients received general anesthesia and were placed in the right lateral decubitus position. Generally, 3 or 4 ports were used. A 10 mm trocar was placed at the lower umbilicus for telescope. The main manipulation 12 mm trocar was placed in the left subcostal midclavicular line and the auxiliary 5 mm trocar was placed at the subxiphoid position. Another 5 mm trocar was placed in the left axillary line for the assistant if necessary. The procedure of anterior or lateral approach LS was detailedly described in the literatures.^[8,9]^ Perisplenic ligaments were dissected with ultrasonic dissector (Harmonic Scalpel, Ethicon Endo-Surgery) or LigaSure vessel sealing system (Covidien/Medtronic, Mansfield, MA), and 3 different methods were used for managing the splenic pedicle, including ligation by snare, secondary pedicle division, and endoscopic linear vascular stapler (Endo-GIA).

For the HALS procedure, patients were placed in a right semi-lateral recumbent position. A subxiphoid midline incision approximately 6 to 8 cm in length was performed, in which the hand port was inserted. The left hand of the surgeon was inserted intraperitoneally through the hand port to help complete the surgery. A 10 mm trocar was placed in the lower umbilicus for telescope. The main manipulation 12 mm trocar was placed at the left midclavicular line below the inferior margin of the spleen. Firstly, gastrocolic ligament and splenogastric ligament were divided using ultrasonic dissector or LigaSure. The lesser sac was opened, and the splenic artery was identified and ligated above the body of the pancreas. Subsequently, splenocolic ligament and splenorenal ligament were dissected. A tunnel behind the splenic hilum was established with the left hand, and the splenic pedicle was handled as related in TLS procedure. Then, the short gastric vessels and splenophrenic ligament were divided. When using snare to management the splenic pedicle, all the perisplenic ligaments should be dissected firstly. Finally, the resected spleen was placed into a retrieval bag and extracted from the midline incision. The peritoneal cavity was irrigated and examined for any active hemorrhage, and a drain was placed in the splenic bed.

### 2.4 Preoperative care and follow-up

Before surgery, the spleen size was estimated in imaging studies (ultrasound/CT/MRI). Patients with ITP received oral prednisolone and immunoglobulin G for 3 to 5 days, starting at least 1 week before LS to increase their platelet counts to 50 × 10^9^/L. Patients with hypersplenism were given a platelet transfusion intraoperatively if the platelet counts less than 50 × 10^9^/L. Antibiotic was routinely given at induction of anesthesia, and continued for several days after surgery depending on the patient's condition. All patients received routine care and postoperative monitoring. Antiplatelet agents (dipyridamole, aspirin) were administrated to prevent thrombus when the platelet count was more than 600 × 10^9^/L. Ultrasonographic screening for portal vein system thrombosis (PVST) was performed on the seventh postoperative day. All patients received every 3 to 6 months follow-up at outpatient clinics or by a telephone interview.

### 2.5 Statistical analysis

The short-term outcomes included operative time, intraoperative blood loss, need for blood transfusions, intraoperative autotransfusion, conversion, reoperation, length of stay, morbidity, and mortality (within 30 days after surgery). The long-term outcomes included respond rate of patients with ITP and esophagogastric variceal bleeding rate with hypersplenism. Data were analyzed with SPSS 22.0 for windows (SPSS, Chicago, IL). Quantitative data were presented as mean ± standard deviation (SD) and compared using Student's *t* test. Qualitative data were presented as number and percentage and compared using chi-square test or Fisher's exact test. Results were statistically significant with *P*-value < .05.

## 3 Results

### 3.1 Patient characteristics

The patients’ characteristics in TLS and HALS group were presented in Table 1. There were no meaningful differences in gender (*P* = .087), hypertension (*P* = .480), diabetes mellitus (*P* = .152), cardiac disease (*P* = .163) between the TLS group and HALS group. Most patients with hematologic benign diseases and splenic benign tumor underwent TLS. However, patients with hypersplenism mostly underwent HALS. One hundred thirty five patients underwent elective LS with additional operation, including 36 TLS with additional operation (TLS plus group) and 99 HALS with additional operation (HALS plus group). The types of additional operation are summarized in Table 2. The most common type of additional operation was esophagogastric devascularization (76.3%, 103/135).

**Table 1:** Patients’ characteristics.

Variables	TLS (n = 222)	HALS (n = 264)	*P*
Gender			.087
Male	108	149	
Female	114	115	
Age (yr)	42.1 ± 14.0	44.5 ± 10.6	.039
Hypertension	67	72	.480
Diabetes mellitus	16	29	.152
Cardiac disease	27	22	.163
Indication			.000
Hypersplenism	96	249	
ITP	69	3	
Other hematologic benign diseases	11	0	
Splenic malignant tumor^∗^	9	5	
Splenic cyst	14	0	
Splenic benign tumor	19	3	
Others	4	4	
Additional operation	36	99	.000
Splenic size (cm)	14.3 ± 4.1	18.5 ± 3.8	.000

HALS = hand-assisted laparoscopic splenectomy, ITP = immune thrombocytopenia, TLS = totally laparoscopic splenectomy.

∗ Incluing splenic malignant lymphoma (n = 11), splenic angiosarcoma (n = 2), and metastatic carcinoma of spleen (n = 1).

**Table 2:** The types of additional operation.

Type of additional operation	TLS (n = 36)	HALS (n = 99)	*P*
Esophagogastric devascularization	24	79	.175
LC	9	10	.055
Partial hepatectomy	0	5	.390
Radiofrequency ablation	1	0	.596
Fenestration of hepatic cyst	1	0	.596
Hernia repair	0	3	.692
Distal pancreatectomy	1	0	.596
LC + esophagogastric devascularization	0	1	.596
LC + partial hepatectomy	0	1	.596

LC = laparoscopic cholecystectomy.

### 3.2 Perioperative outcomes

Three methods were used to manage the spleen pedicle in our series (except conversion), including ligation by snare (n = 21), secondary pedicle division (n = 34), and Endo-GIA (n = 422). The mean operative time, intraoperative blood loss, and length of stay were 149.4 ± 63.3 minutes, 230.1 ± 225.1 mL, and 6.7 ± 3.2 days, respectively. Perioperative outcomes of 4 subgroups (TLS, TLS plus, HALS, and HALS plus) were listed in Table 3. Among elective LS without additional operation, there were no difference in intraoperative blood loss (*P* = .287), intraoperative autotransfusion rate (*P* = .134), conversion (*P* = .375), reoperation (*P* = .288), and morbidity (*P* = .442) between TLS and HALS group; TLS group exhibited a longer operative time (*P* = .000), but had a lower intraoperative blood transfusion rate (*P* = .000) and a shorter postoperative hospital stay (*P* = .000), compared with HALS group. Among elective LS with additional operation, TLS plus and HALS plus group were comparable in intraoperative autotransfusion rate (*P* = .224), conversion (*P* = .118), reoperation (*P* = 1.000), morbidity (*P* = .695), and postoperative hospital stay (*P* = .110); TLS plus group exhibited a longer operative time (*P* = .000), more intraoperative blood loss (*P* = .014), and higher intraoperative blood transfusion rate (*P* = .013), compared with HALS plus group. Nine patients (1.9%, 9/486) were converted to open surgery. The cause of conversion was bleeding (n = 7), needing distal pancreatectomy (n = 1), and needing partial hepatectomy (n = 1). There were 101 (21.0%) patients who underwent intraoperative autotransfusion. Two (0.4%, 2/486) patients required reoperation because of postoperative hemorrhage. There was no perioperative death.

**Table 3:** Perioperative outcomes of 4 groups.

	Without			With		
Variables	TLS (n = 186)	HALS (n = 165)	*P*	TLS plus (n = 36)	HALS plus (n = 99)	*P*
Operative time (min)	156.4 ± 67.7	126.9 ± 56.5	.000	198.4 ± 47.6	157.1 ± 57.3	.000
Intraoperative blood loss (mL)	230.2 ± 215.5	204.4 ± 239.7	.287	336.9 ± 218.7	233.5 ± 210.9	.014
Need for blood transfusion (n)	61 (32.8%)	87 (52.7%)	.000	24 (66.7%)	42 (42.4%)	.013
Intraoperative autotransfusion (n)	33 (17.7%)	40 (24.2%)	.134	10 (27.8%)	18 (18.2%)	.224
Conversion	3 (1.6%)	1 (0.6%)	.375	3 (8.3%)	2 (2.0%)	.118
Reoperation	0	1 (0.6%)	.288	0	1 (1.0%)	1.000
Morbidity	25 (13.4%)	26 (15.8%)	.442	15 (41.7%)	45 (45.5%)	.695
Mortality	0	0	NA	0	0	NA
Length of stay (d)	5.4 ± 2.5	7.6 ± 3.2	.000	6.6 ± 3.8	7.7 ± 3.4	.110

Accessory spleen was found in 46 (9.5%, 46/486) patients. The location of accessory spleen included splenic hilum (n = 22), greater omentum (n = 8), gastrosplenic ligament (n = 6), splenocolic ligament (n = 5), pancreatic tail (n = 3), and small bowel mesentery (n = 2). Accessory spleens were resected together with the spleen in patients with hematologic benign diseases and splenic malignant tumor.

As shown in Table 4, 112 (23.0%) patients occurred postoperative complications, including 96 PVST, 4 postoperative hemorrhage, 4 fever of unknown origin, 2 pulmonary infection, 1 pleural effusion, 1 pancreatic fistula, 1 spontaneous peritonitis, 1 subphrenic hematoma, 1 subphrenic abscess, 1 port-site bleeding, and 2 left lateral abdominal wall diffuse ecchymosis. There were 2 patients occurred more than 1 type of postoperative complication, including 1 case with PVST and postoperative hemorrhage, and 1 case with PVST and pulmonary infection. The incidence of PVST in HALS was higher than that in TLS (23.9% vs 14.9%, *P* = .013). According to the Clavien-Dindo classification, 6 patients were grade I, 100 grade II, 4 grade IIIa, and 2 grade IIIb.

**Table 4:** Postoperative morbidity.

Complications	TLS (n = 40)	HALS (n = 72)	*P*
Portal venous system thrombosis	33	63	.013
Postoperative hemorrhage	1	3	.629
Fever of unknown origin	1	3	.629
Pulmonary infection	0	2	.503
Pleural effusion	1	0	.457
Pancreatic fistula	1	0	.457
Spontaneous peritonitis	0	1	1.000
Subphrenic hematoma	1	0	.457
Subphrenic abscess	0	1	1.000
Port-site bleeding	0	1	1.000
Left lateral abdominal wall diffuse ecchymosis	2	0	.208

### 3.3 Anterior versus lateral approach in TLS

In TLS, 63 (28.4%) patients underwent the lateral approach and 159 (71.6%) patients underwent the anterior approach. As shown in Table 5, there was no significant difference in operation time (*P* = .537), intraoperative blood loss (*P* = .919), conversion rate (*P* = 1.000), morbidity (*P* = .802), and postoperative hospital stay (*P* = .204) between the 2 approaches.

**Table 5:** Anterior versus lateral approach in TLS.

	TLS		
Variables	Lateral approach (n = 63)	Anterior approach (n = 159)	*P*
Operative time (min)	158.8 ± 68.9	165.0 ± 65.9	.537
Intraoperative blood loss (mL)	245.2 ± 192.9	248.6 ± 229.2	.919
Need for blood Transfusion (n/%)	19 (30.2%)	66 (41.5%)	.117
Conversion (n/%)	2 (3.2%)	4 (2.5%)	1.000
Reoperation (n)	0	0	NA
Morbidity (n/%)	12 (19.0%)	28 (17.6%)	.802
Mortality (n)	0	0	NA
Length of stay (d)	6.0 ± 3.3	5.5 ± 2.5	.204

### 3.4 Perioperative outcomes of TLS and HALS for splenomegaly > 17 cm

The perioperative outcomes of TLS and HALS (without additional operation) for splenomegaly (maximum diameter greater than 17.0 cm) were listed in Table 6. HALS exhibited a shorter operative time (114.4 ± 49.5 vs 205.5 ± 65.3 minutes, *P* = .000), and less intraoperative blood loss (160.4 ± 197.7 vs 357.3 ± 260.3 mL, *P* = .000) compared to TLS. The conversion rate (4.4% vs 1.2%, *P* = .271) and morbidity (13.3% vs 14.0%, *P* = .394) of HALS were comparable with that of TLS. However, the postoperative hospital stay of HALS was significantly longer than that of TLS (7.6 ± 3.1 vs 6.2 ± 2.8 days, *P* = .014). No reoperation and mortality were observed in both groups.

**Table 6:** Perioperative outcomes for splenomegaly > 17 cm.

Variables	TLS (n = 45)	HALS (n = 86)	*P*
Splenic size (cm)	19.6 ± 1.6	20.8 ± 3.1	.004
Operative time (min)	205.5 ± 65.3	114.4 ± 49.5	.000
Intraoperative blood loss (mL)	357.3 ± 260.3	160.4 ± 197.7	.000
Need for blood Transfusion (n/%)	23 (51.1%)	40 (46.5%)	.617
Conversion (n/%)	2 (4.4%)	1 (1.2%)	.271
Reoperation (n)	0	0	NA
Morbidity (n/%)	6 (13.3%)	12 (14.0%)	.922
Mortality (n)	0	0	NA
Length of stay (d)	6.2 ± 2.8	7.6 ± 3.1	.014

### 3.5 Outcomes of follow-up

Up to December 2019, 72 patients with ITP were followed-up for 2 to 181 months (average: 89.5 months). Response to splenectomy was assessed at the last available follow-up. According to the criteria of the International Working Group endorsed by the American Society of Hematology guidelines,^[10,11]^ 47 (65.3%) patients achieved complete response (CR), 9 (12.5%) response (R), 16 (22.2%) no response (NR). The total therapeutic response (CR + R) rate was 77.8% (56/72).

Among 345 patients with hypersplenism, 25 (7.2%) patients were lost to follow-up, the other 320 (92.8%) patients were follow-up for 10 to 160 months (average: 71.9 months). Theirs’ white blood cell and platelet counts were all rose to above normal level after the operation. During the follow-up period, esophagogastric variceal bleeding (EGVB) recurred in 22 (6.9%) patients. All these patients underwent endoscopic therapy, and 3 patients died from acute upper digestive tract rebleeding. Ten (3.1%) patients occurred secondary liver cancer, and 4 died from secondary liver cancer.

## 4 Discussion

Since its introduction in 1991, LS has gained worldwide acceptance with many advantages over OS. With the development of laparoscopic techniques and instruments, the indication of LS had gradually expanded from normal size spleen to massive splenomegaly. Although LS was a routine procedure in many centres, only few reports with more than 300 cases.^[12–15]^ The reported morbidity following LS varied from 0% to 35.7%, with mortality varied from 0% to 3.9%, the conversion rate varied from 0% to 4%, and the reoperation rates varied from 0% to 6.7% in the literatures.^[16]^ In November 2002, our team firstly performed LS for a 35-years old woman with ITP. Since then, the volumes of LS were gradually increased in our institution, especially after the introduction of HALS in 2006. During the past 16 years, our indications of LS have broadened from benign hematological disorders to massive even supermassive splenomegaly secondary to hepatic cirrhosis. Meanwhile, the procedure has expanded from single LS to LS combination with esophagogastric devascularization. Herein, we presented the short- and long-term outcomes of 486 elective LS. In our series, the mean operative time, intraoperative blood loss, and length of stay were 149.4 minutes, 230.1 mL, and 6.7 days, respectively. The morbidity, mortality, reoperation, and conversion rate were 22.8%, 0, 0.4%, and 1.9%, respectively. Obviously, these perioperative outcomes were consistent with those reported by the literatures.

In regard to surgical techniques, safely dissecting the perisplenic ligments and managing the spleen pedicle are the most important manipulations for successful LS. Usually, there have 2 surgical approaches for LS, including anterior approach and lateral approach. The lateral approach provides better exposure of the splenic hilum and the pancreatic tail because the abdominal viscera are retracted away from the upper-left quadrant by gravity, allowing easier dissection of the splenic hilar structures. Recently, a systematic review and meta-analysis suggested that lateral approach is superior to anterior approach with the advantage of better access, more secure hemostasis, less conversion to open surgery, less morbidity, earlier recovery, and shorter length of hospital stay.^[17]^ In our opinion, there were no significant difference between the 2 approaches, and the choice of approach should be depended on the surgeon's experience and concomitant conditions. Three methods were used to manage the spleen pedicle in our series, and they have their own advantage and disadvantage. Endo-GIA transection is a simple, effective, and time-saving method, but it is more expensive than other 2 methods. Ligation by snare is an economical and effective method; however, it needs to completely dissect the perisplenic ligments and fully mobilize the spleen, which is difficult under the circumstance of perisplenic adhension and massive splenomegaly. Secondary pedicle division strategy is a highly cost-effective method. Nevertheless, its disadvantages are technique challenging and higher risk of bleeding. Whatever method is used, we recommended to ligate the splenic artery in patients with splenomegaly as early as possible, which could decrease the volume of spleen and lower the risk of massive hemorrhage during operation.

Hypersplenism is a clinical syndrome characterized by an enlarged, overactive spleen. Currently, splenectomy and partial splenic embolization (PSE) are the most popular treatment for hypersplenism. LS can eliminate hypersplenism-induced blood cell destruction, prevent EGVB, and decrease portal pressure and reverse hypersplenism.^[18]^ As a non-surgical intervention, PSE is an effective option for patients who are not surgical candidates. PSE owns several advantages over conventional LS including decreased the incidence of PVST and preservation of splenic tissue function to protect against infections.^[19]^ However, the effect of PSE is strongly dependent on the infarcted splenic volume, a relative insufficient embolization extent may lead to the recurrence of hypersplenism; and this procedure still has a high risk and can cause substantial complications.^[20]^ At present study, the most frequent indication was hypersplenism secondary to hepatic cirrhosis (71.0%). All patients’ white blood cell and platelet counts were all rose to above normal level after LS. The EGVB rate was 6.9% after a mean 71.9 months follow-up. Therefore, the clinical effect of LS for hypersplenism was verified in our series.

As we known, LS for hypersplenism has always been a technique challenging due to the limited working space, increased risk of bleeding, the potential risk of increasing conversion, operative time, and morbidity. Therefore, there is some controversy regarding laparoscopic operations of markedly enlarged spleens and patients with portal hypertension. Some studies supported the laparoscopic approach, clearly demonstrating the benefits of LS even in the case of massively enlarged spleens. This is very well documented in the system review published by Cai et al^[21]^ comparing laparoscopic to open splenectomy for portal hypertension. However, portal hypertension caused by liver cirrhosis is considered as a contraindication for LS in the clinical practice guidelines of the European Association for Endoscopic Surgery (EAES). It also recommends HALS for massive splenomegaly to avoid conversion to OS and complications.^[4]^ Targarona et al^[22]^ conducted a comparison between conventional LS and HALS for splenomegaly (final spleen weight > 700 g) and concluded that HALS was associated with shorter operative times, less morbidities, and shorter hospital stays. Wang et al^[23]^ compared the outcomes of TLS (n = 20) and HALS (n = 19) for splenomegaly (maximum diameter greater than 17 cm) and hypersplenism due to cirrhosis, the results showed that TLS had a longer operative time, more estimated blood loss, more patients requiring transfusion, and more complications than HALS. Recently, a meta-analysis of HALS versus LS for splenomegaly showed that the operative time was significantly shorter, blood loss volume and conversion rate were significantly lower in the HALS group than those in the LS group. However, no significant difference was observed in hospital stay length, blood transfusion, time to food intake, complications, or mortality rate between the 2 groups.^[24]^ Based on our results, we recommended HALS for splenomegaly > 17 cm because of HALS with a shorter operative time, less intraoperative blood loss, and comparable conversion rate and morbidity. Furthermore, HALS was convenient for extracting the enlarged spleen and performing esophagogastric devascularization for patients with esophageal and gastric varices.

Accessory spleens represent the most common anatomic abnormality and are present in 15% to 30% of children. They most likely originate from mesenchymal remnants that do not fuse with the main splenic mass. Accessory spleens are most commonly located medial to the splenic hilum, adjacent to or within the pancreatic tail or in the splenorenal ligament. Rarely they may be located elsewhere in the abdomen. Surgeons must be cognizant of these locations and routinely check for their presence at the time of planned total splenectomy, because a missed accessory spleen can lead to recurrence of ITP or hereditary spherocytosis. There were several reports of laparoscopic accessory splenectomy after initial splenectomy in the management of recurrent hematologic diseases.^[25,26]^ At present study, accessory spleen was found in 9.5% patients and resected together with the spleen in patients with hematologic benign diseases and splenic malignant tumor. No patient had recurrence related to accessory spleen.

Therapy-resistant ITP was the second frequent indication of LS in our series. Splenectomy is recommended as the mainstay second-line treatment for adult ITP. The indications include: the patient has failed glucocorticoid treatment and has been diagnosed with ITP for more than 6 months; the maintenance dose of prednisone exceeds 15 mg/day, or the patient has a contraindication to glucocorticoids.^[27]^ It is reported that about 80% of ITP patients respond to splenectomy, and 66% of patients can sustain the response with no additional therapy for more than 5 years.^[28,29]^ LS can provide better short-term results, such as less blood loss and blood transfusion, quicker resumption of oral intake, shorter hospital stays, and comparable long-term results to those of OS for ITP.^[30,31]^ Tastaldi et al^[32]^ reported 109 patients with ITP underwent LS, the initial response rate, and long-term response rates at a median 62 months follow-up were 90.8% and 68%, respectively. They also found that there was no statistically significant difference when LS performed before or after second-line medical therapies, and only a robust increase in platelet counts on short-term being associated with long-term response. Xu et al^[33]^ presented a retrospective analysis of 114 patients with ITP who underwent LS from 2001 to 2013. The 140-month response rate to LS was 68%. Multivariate analysis showed that age and postoperative platelet count were independently associated with response rate. Among those 72 patients with ITP in our study, CR was found in 65.3% and R occurred in 12.5% of the cases within a mean follow-up of 89.5 months; that is, a total of 77.8% of the patients responded to splenectomy performed in accordance with the guidelines.

PVST was the most frequent complications in our series with the incidence of 19.8%, which accounted for 85.7% of the overall morbidity. Recent reports have described an incidence of PSVT after LS > 50%.^[34]^ It is reported that massive splenomegaly, splenic weight, and splenic vein diameter are independent risk factors for PSVT after LS.^[35–37]^ Our results showed that the incidence of PVST in HALS group was significantly higher than that in TLS group, probably because there were more patients with hypersplenism in HALS group. Although mostly patients with PVST following LS were asymptomatic, its high incidence justifies ultrasonographic screening on the seventh postoperative day. For patients at high risk, we suggested that the usage of low-molecular-weight heparin within the first 5 days after surgery, followed by oral warfarin or aspirin for 1 month for the prevention of PVST. Postsplenectomy thrombocytosis may increase the risk of thromboembolism, especially in patients with hematological malignancies or myeloproliferative disorders. It is necessary for surveilling the platelet counts after LS, and we recommend a threshold of 600 × 10^9^/L to begin antiplatelet agents (dipyridamole, aspirin).

There were some limitations for the present study. First of all, it was a retrospective study; therefore, selection bias may unavoidable in this study. Second, the choice of either TLS or HALS was mainly based on the surgeon's judgement. Several different surgeons performed the LS in our institution, which may have had an impact on the results of the study. Third, this study is based on a single-center experience, which may lead to the lack of representativeness. Therefore, a multi-center, prospective, and randomized study is necessary for further study.

## 5 Conclusion

In conclusion, our results showed LS is a safe, feasible, and effective procedure with satisfactory short- and long-term outcomes. HALS is an alternative technique in patients with massive spleens.

## Author contributions

**Conceptualization:** Xiaowei Fu, Yong Li, Weidong Xiao.

**Data curation:** Zhengjiang Yang, Shuju Tu, Wanpeng Xin, Haiming Chen, Xueming Li.

**Formal analysis:** Xiaowei Fu, Zhengjiang Yang, Weidong Xiao.

**Funding acquisition:** Weidong Xiao

**Investigation:** Zhengjiang Yang, Shuju Tu, Wanpeng Xin.

**Software:** Xiaowei Fu, Zhengjiang Yang.

**Supervision:** Yong Li, Weidong Xiao.

**Writing – original draft:** Xiaowei Fu, Weidong Xiao.

**Writing – review & editing:** Weidong Xiao.
